# Soft robotic platform for controlled, progressive and reversible aortic constriction in a small animal model

**DOI:** 10.21203/rs.3.rs-3100659/v1

**Published:** 2023-07-19

**Authors:** Luca Rosalia, Sophie X. Wang, Caglar Ozturk, Wei Huang, Jean Bonnemain, Rachel Beatty, Garry P. Duffy, Christopher T. Nguyen, Ellen T. Roche

**Affiliations:** 1 Health Sciences and Technology Program, Harvard University - Massachusetts Institute of Technology, Cambridge, 02139-4307, MA, USA.; 2 Institute for Medical Engineering and Science, Massachusetts Institute of Technology, Cambridge, 02139-4307, MA, USA.; 3 Department of Surgery, Beth Israel Deaconess Medical Center, Boston, 02215, MA, USA.; 4 Koch Institute For Integrative Cancer Research, Massachusetts Institute of Technology, Cambridge, 02142, MA, USA.; 5 Department of Adult Intensive Care Medicine, Lausanne University Hospital, Lausanne, 1011, Switzerland.; 6 Anatomy and Regenerative Medicine Institute, College of Medicine Nursing and Health Sciences, University of Galway, Ireland, Galway, H91 W2TY, Ireland.; 7 Department of Cardiovascular Medicine, Radiology, and Biomedical Engineering, Cleveland Clinic, Cleveland, 44195, OH, USA.; 8 Department of Mechanical Engineering, Massachusetts Institute of Technology, Cambridge, 02139, MA, USA.

## Abstract

Our understanding of cardiac remodeling processes due to left ventricular pressure overload derives largely from animal models of aortic banding. However, these studies fail to simultaneously enable control over disease progression and reversal, hindering their clinical relevance. Here, we describe a method for controlled, progressive, and reversible aortic banding based on an implantable expandable actuator that can be finely controlled to modulate aortic banding and debanding in a rat model. Through catheterization, imaging, and histologic studies, we demonstrate that our model can recapitulate the hemodynamic and structural changes associated with pressure overload in a controllable manner. We leverage the ability of our model to enable non-invasive aortic debanding to show that these changes can be partly reversed due to cessation of the biomechanical stimulus. By recapitulating longitudinal disease progression and reversibility, this model could elucidate fundamental mechanisms of cardiac remodeling and optimize timing of intervention for pressure overload.

## Introduction

Left ventricular (LV) pressure overload plays a key role in the onset of heart failure with preserved ejection fraction (HFpEF), which accounts for approximately 50% of all cases of heart failure [1, 2]. This biomechanical state is typically induced by conditions which cause an increase in the afterload, such as aortic stenosis (AS) and hypertension [3, 4]. HFpEF often arises as a result of remodeling processes resulting from LV pressure overload, which induce stiffening of the LV wall and lead to the inability of the heart to fill adequately and therefore eject enough blood to meet the metabolic demands of the body [5, 6]. Treatments for patients with HFpEF are currently limited, with scarce medical therapy options and no HFpEF-specific FDA-approved devices [7]. The lack of therapeutics for these patients is largely due to our incomplete understanding of fundamental processes driving cardiac remodeling in HFpEF and of the plasticity and potential reversibility of these processes [8].

Aortic banding is a surgical technique involving partial ligation of the aorta which is broadly used to study cardiac remodeling processes due to pressure overload associated with HFpEF in preclinical models [9, 10]. The literature counts hundreds of studies of aortic banding across a variety of animal species, reporting on several nuances in the surgical approach and techniques for structural and functional evaluation. Consistently, all the methods developed to date fail to enable control over the severity of aortic constriction and, simultaneously, over the dynamics of disease progression and reversal, hindering fundamental and translational studies of human disease. In large animal models, aortic banding has been previously achieved through a variety of methods [11], including use of an inelastic sleeve in a juvenile swine that becomes constrictive during the animal’s development [12], an on-demand inflatable cuff that can be pressurized to various levels [13], and a reduction stent that can be delivered percutaneously in the descending thoracic aorta [14]. Compared to large animals, small animal models are more time- and cost-effective due to the relatively faster dynamics of their associated cardiac remodeling processes. Therefore, they lend themselves to the development of multi-hit studies where aortic banding is combined with other comorbidities of HFpEF, and represent the most commonly used model in this field [15]. For these reasons, this work will focus on small animal models of aortic banding.

In small animals, aortic banding is performed either at the thoracic or the abdominal level. In turn, thoracic aortic banding can be done either at the ascending aorta (ascending aortic constriction, AAC) or at the aortic arch between the brachiocephalic and the left carotid arteries (transverse aortic constriction, TAC); whereas abdominal banding involves suprarenal aortic constriction (SAC) above the origin of both renal arteries [16–18]. While being the most challenging to perform from a surgical standpoint, AAC is the most relevant model of pressure overload due to AS and secondary HFpEF [15, 19]. This is because, unlike the TAC and SAC approaches, AAC does not induce pressure overload in the brachiocephalic artery or in the entire upper body circulation, and is therefore more representative of the hemodynamics of AS compared to other techniques.

Different degrees of aortic banding can result in various levels of cardiac remodeling and diastolic dysfunction [20, 21]. The degree of constriction can be coarsely controlled by choice of the size of the needle used during ligation, which determines how tight the knot around the aorta will be [22]. An alternative approach involves implantation of o-rings or clips with varying inner diameter dimensions [23–25]. Neither of these techniques, however, enables temporal control over the degree of aortic banding and therefore fail to re-create pathophysiological processes of disease onset and progression that are clinically relevant. For example, implantation of a loose ring (or use of a large needle) results in a relatively mild degree of pressure overload, which fails to follow the natural progression observed over time in humans due to disease exacerbation. Contrarily, implantation of a tight ring (or small needle) causes an abrupt onset of severe pressure overload which is rarely observed in patients. Another method, known as intermittent banding, involves tunneling of the suture to the back of the animal so that it can be pulled to induce intermittent pressure overload [26]. While this technique could capture cardiac remodeling processes triggered by intermittent stimuli, such as during exercise, it does not provide significant insights into pathophysiological processes associated with disease.

A major limitation of current aortic banding small animal models is their inability to reverse pressure overload. Reversal is crucial for studies of ventricular remodeling plasticity, which would advance our fundamental understanding of disease progression and enable direct study of the impact of therapeutics [14, 27]. Unfortunately, current approaches require surgical removal of the suture or clip through an additional intervention, increasing animal morbidity and mortality rates associated with these studies [25, 28–30]. Altogether, limitations in these techniques highlight the need for an approach that enables controllability of the degree of aortic banding and modulation of the dynamics of disease progression, and that provides a less-invasive means to reverse pressure overload.

In previous work, we demonstrated the ability of soft robotic tools to recapitulate the hemodynamics of AS in a benchtop and a sub-acute swine model [31, 32]. Here, we describe the use of soft robotics for the development of a highly controllable chronic small animal model of aortic banding that overcomes the shortcomings of other techniques. We leverage invasive hemodynamic monitoring to evaluate clinical metrics of cardiac function and AS in the acute setting. Uniquely, we use magnetic resonance imaging (MRI) to visualize aortic flow resulting from aortic banding, and to characterize the degree of aortic constriction, cardiac remodeling, and reversal in a chronic study.

## Results

### Soft robotic actuator enables controlled banding and debanding in a rat model

**Fig. 1 |:**
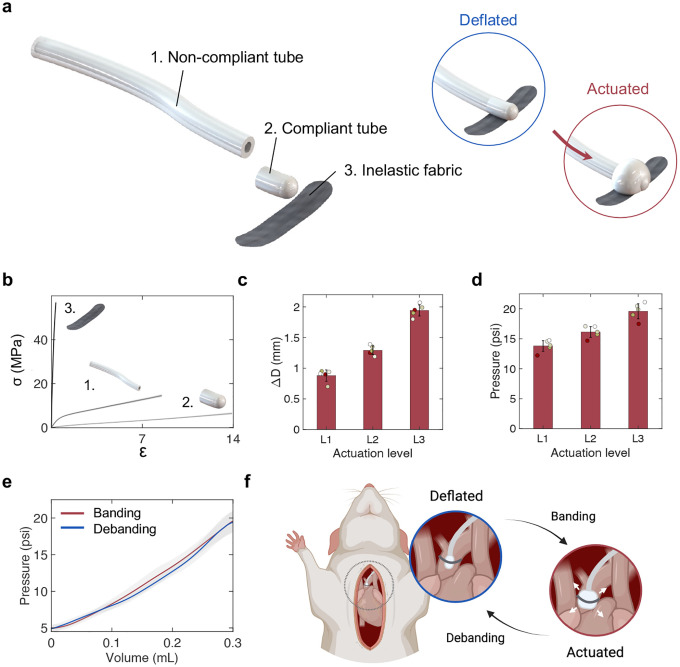
Design overview and mechanical characterization of soft robotic actuator. **a,** Components of the soft robotic actuator, highlighting the non-compliant and compliant tubings and the inelastic fabric sheet. Detail of the compliant tubing in the deflated and actuated states. **b,** Stress-stress behavior of the materials constituting the actuator (n = 5 for each material). **c-d,** Maximum diameter (**c**) and pressure (**d**) at three distinct actuation levels (L1-L3). **e,** Pressure-volume curves during banding and debanding. **f,** Illustration of the actuator positioned around the ascending aorta of a rat model. Detail of the expansion of the implanted actuator during aortic banding and debanding. Data show mean ± 1 S.D. Each test was conducted on n = 5 actuators and repeated n = 5 times.

We designed and manufactured a soft robotic actuator composed of a non-compliant (ID = 1/32”, OD = 3/32”, silicone rubber) and a compliant (ID = 1/32”, OD = 1/16”, PVC) tubing and an inelastic fabric sheet (Fig. 1a). Under pressure, the compliant tube expands from a deflated to an actuated state (Fig. 1a). The non-compliant tube prevents expansion of the actuator beyond the compliant material and the inelastic sheet allows expansion in one direction only, away from the fabric. The compliant tubing is connected to the non-compliant tube through a carbon fiber tube (ID = 0.02”, OD = 0.04”) wrapped in a heat-shrink tube that reinforces the connection between the two tubings. A silicone rubber adhesive seals the other end of the compliant material. The uniaxial tensile response of these materials (Fig. 1b) highlights differences in their Ultimate Tensile Strength (UTS_1_ = 15.2 ± 0.5 MPa; UTS_2_ = 6.9 ± 0.3 MPa; UTS_3_ = 79.2 ± 4.2 MPa), Young’s modulus (E_1_ = 7.5 ± 0.1 MPa; E_2_ = 0.83 ± 0.05 MPa; E_3_ = 135.3 ± 5.3 MPa) and maximum elongation ($\epsilon_{1}^{max}=9.1\pm 0.6$; $\epsilon_{2}^{max}=15.0\pm 1.2$; $\epsilon_{3}^{max}=0.8\pm 0.1$).

We quantified the radial expansion of the compliant tube for three different actuation levels (L1–3), achieved by injecting distinct volumes of glycerin as the actuation medium, namely L1 = 0.26 mL, L2 = 0.28 mL, L3 = 0.3 mL. These volumes were used to recreate (and progress between) distinct severities of pressure overload in in vivo experiments. Changes in maximal diameter for the three levels are reported in Fig. 1c (ΔD_*L*1_ = 0.88 ± 0.09 mm; ΔD_*L*2_ = 1.29 ± 0.07 mm; ΔD_*L*3_ = 1.94 ± 0.09 mm). Fig. 1d shows the corresponding maximum pressure measured inside the actuator at the three levels of actuation (P_*L*1_ = 13.8 ± 0.9 psi; P_*L*2_ = 16.1 ± 0.9 psi; P_*L*3_ = 19.6 ± 1.3 psi). The pressure-volume characteristics of the actuator during continuous banding and debanding at constant-rate (0.3 mL/s) infusion and withdrawal of up to 3 mL of medium (n = 5) are shown in Fig. 1e.

The design and the behavior of the device were optimized to enable in vivo implantation around the ascending aorta of the animals, allowing for controlled, progressive, and reversible banding. The positioning of the device on the ascending aorta and changes to the diameter of the actuator during banding and debanding are illustrated in Fig. 1f.

### Modulation of cardiac hemodynamics at distinct levels of aortic banding and debanding

**Fig. 2 |:**
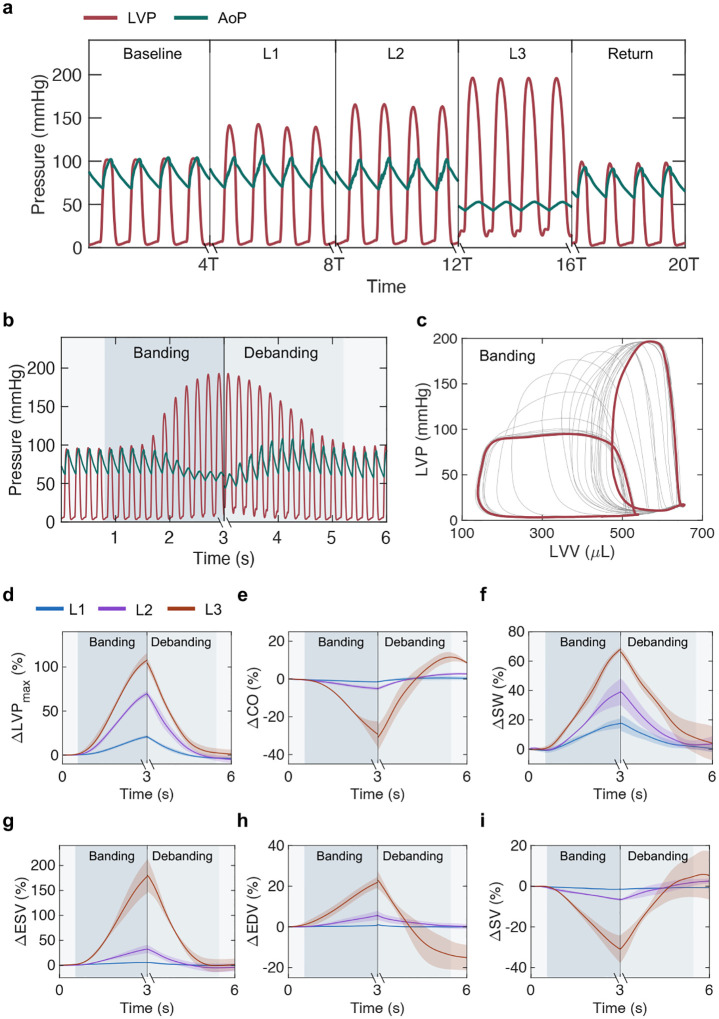
**Modulation of cardiac hemodynamics during acute aortic banding and debanding in catheterization study. a,** Representative LVP and AoP waveforms at baseline, L1-L3 and return to baseline following debanding. **b,** Representative progression of LVP and AoP during banding and debanding. **c,** Changes in the LV PV loop during banding. Highlighted loops correspond to baseline and peak banding. **d-i,** Relative changes in cardiac function during banding and debanding for three actuation levels (L1-L3), including LVP_*max*_ (**d**), CO (**e**), SW (**f**), ESV (**g**), EDV (**h**), and SV (**i**). Data show mean ± 1 S.D. Each test was conducted on n = 5 animals and repeated n = 3 times. LVP = left ventricular pressure; AoP = aortic pressure; T = heart cycle period; LVV = left ventricular volume; CO = cardiac output; SW = stroke work; ESV = end-systolic volume; EDV = end-diastolic volume; SV = stroke volume.

We demonstrated the controllability of the actuator in an acute rat model through invasive hemodynamic monitoring of LV function. Fig. 2a shows representative LV pressure (LVP) and aortic pressure (AoP) waveforms over four consecutive heartbeats at baseline, three distinct levels of actuation (L1-L3) and at return to baseline after debanding. Elevations in LVP and a reduction in the amplitude (pulse) of the AoP are consistent with hemodynamic changes associated with pressure overload. Through constant-rate volume-control pressurization of the soft robotic actuator, we could visualize progressive hemodynamic changes during banding and debanding. Changes in LVP and AoP over actuation are shown in Fig. 2b. Analogously, the progression of the LV pressure-volume (PV) loop during banding is depicted in Fig. 2c, highlighting elevations in LVP and a drop in the stroke volume (SV) due to banding.

Fig. 2 d–i show progressive changes in parameters of LV function for each actuation level during both banding and debanding. As expected, aortic banding resulted in an increase in LVP_*max*_ ($\Delta {\text{LVP}}_{L1}^{max}=21.0\pm 1.7\;\%$; $\Delta {\text{LVP}}_{L2}^{max}=69.7\pm 3.3\;\%$; $\Delta {\text{LVP}}_{L3}^{max}=107.6\pm 8.2\;\%$, Fig. 2d), a drop in cardiac output (ΔCO_*L*1_ = −1.5 ± 0.1 %; ΔCO_*L*2_ = −5.1 ± 0.9 %; ΔCO_*L*3_ = −29.3 ± 6.9 %, Fig. 2e), and a raise in stroke work (ΔSW_*L*1_ = 17.7 ± 3.3 %; ΔSW_*L*2_ = 39.1 ± 9.0 %; ΔSW_*L*3_ = 68.2 ± 1.7 %, Fig. 2f). Due to banding, we also observed an increase in the end-systolic volume (ΔESV_*L*1_ = 5.7 ± 0.9 %; ΔESV_*L*2_ = 32.6 ± 8.2 %; ΔESV_*L*3_ = 178.1 ± 33.6 %, Fig. 2g) and in the end-diastolic volume (ΔEDV_*L*1_ = 0.8 ± 0.2 %; ΔEDV_*L*2_ = 5.7 ± 2.3 %; ΔEDV_*L*3_ = 21.9 ± 2.7 %, Fig. 2h), leading to an overall drop in SV (ΔSV_*L*1_ = −1.4 ± 0.3 %; ΔSV_*L*2_ = −6.5 ± 0.6 %; ΔSV_*L*3_ = −31.0 ± 6.7 %, Fig. 2i).

In this acute demonstration of our platform, all metrics of cardiac function (Fig. 2 d–i) were calculated at peak banding, before physiologic compensation to pressure overload. Debanding was then performed 3−5 seconds after peak banding. Although all metrics showed a reversal of the acute changes induced by aortic constriction standard deviations were generally higher in the return state than at baseline. These changes were particularly significant following the most severe level of banding (L3), likely due to differences in the tolerance of severe aortic constriction among different animals.

### MRI and catheterization studies show recapitulation of the hemodynamics of AS and reversal

**Extended Data Fig. 1 |:**
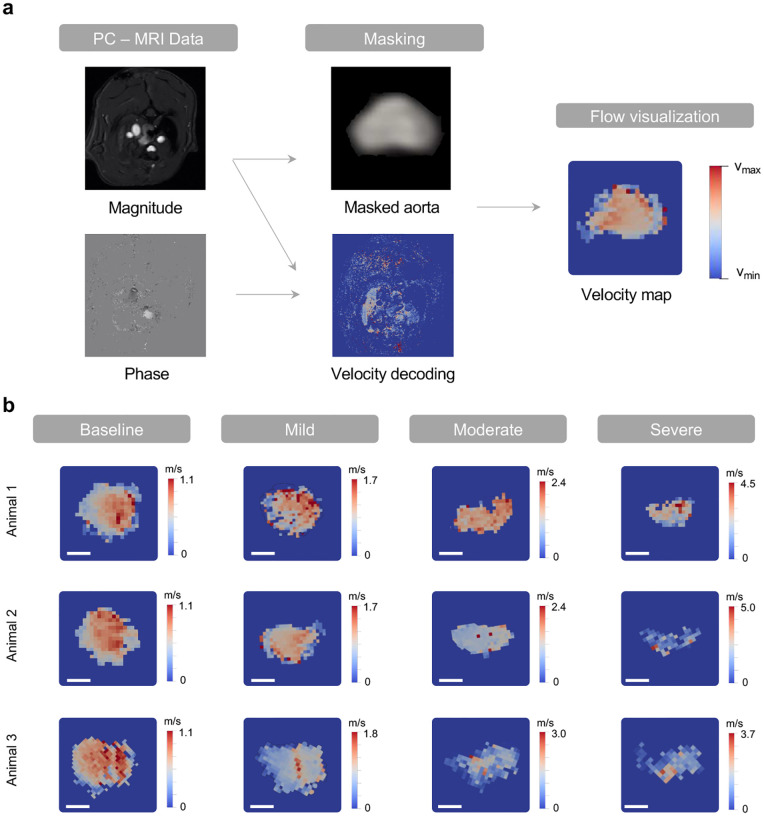
MRI analysis of aortic flow velocity. **a,** Overview of aortic flow MRI analysis. Phase-contrast magnitude and phase data are processed to generate aortic masks and a velocity decoding map. The overlay between the mask and the map enables aortic flow visualization. **b,** 2D aortic flow velocity maps for n = 3 animals at baseline and for mild, moderate, and severe aortic constriction. Scale bar = 1 mm.

**Fig. 3 |:**
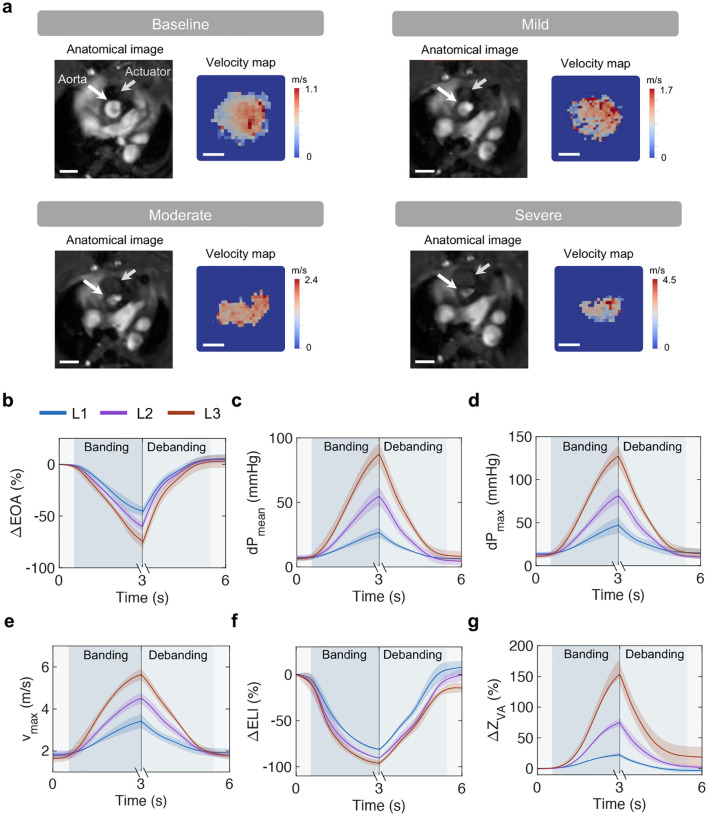
Aortic hemodynamics during acute aortic banding and debanding. **a,** Representative cross-sectional images of the actuator and aorta with corresponding 2D aortic velocity maps at baseline and distinct actuation levels. Scale bar = 3 mm on anatomical images and 1 mm on velocity maps. **b-g,** Changes in aortic hemodynamics measured via catheterization during banding and debanding for three actuation levels (L1-L3), including EOA_*max*_ (**b**), dP_*mean*_ (**c**), dP_*max*_ (**d**), v_*max*_ (**e**), ELI (**f**), Z_*V A*_ (**g**). Data show mean ± 1 S.D. Each test was conducted on n = 5 animals and repeated n = 3 times. EOA = effective orifice area; dP = transvalvular pressure gradient; v = blood flow velocity; ELI = energy loss index; Z_*V A*_ = valvulo-arterial impedance.

We visualized the aortic cross-section and characterized changes in the blood velocity profile using magnetic resonance imaging (MRI). Extended Fig. 1a summarizes the analysis workflow from phase-contrast (PC) MRI data to allow for flow visualization on 2D velocity maps. Fig. 3a illustrates the soft robotic actuator and aortic cross-sections and corresponding 2D velocity maps at baseline and for mild, moderate, and severe banding. Images show that the actuator progressively constricts the aorta at increasing levels of banding. Correspondingly, the peak blood flow velocity at the constriction increased (from *v*_*max*_ = 1.1 m/s at baseline to *v*_*max*_ = 4.5 m/s at severe constriction) as a result of progressive banding. The 2D aortic velocity profiles due to acute aortic banding on n = 3 animals are shown in extended data Fig. 1b.

Changes in the orifice area, peak blood flow velocity, and aortic hemodynamics due to banding and debanding for L1-L3 were comprehensively characterized using catheterization techniques (Fig. 3 b–g). For this approach, we inserted two PV catheters into the LV, advancing one into the aortic arch through the area of aortic constriction. At the three levels of banding (L1-L3), we observed a drop in the effective orifice area (ΔEOA_*L*1_ = −45.0 ± 4.5 %; ΔEOA_*L*2_ = −60.1 ± 4.2 %; ΔEOA_*L*3_ = −74.2 ± 6.0 %, Fig. 3b), an increase in the transvalvular pressure gradient (${\text{dP}}_{L1}^{\text{mean}}=26.6\pm 4.0\text{ mmHg}$; ${\text{dP}}_{L2}^{\text{mean}}=54.7\pm 7.2\text{ mmHg}$; ${\text{dP}}_{L3}^{\text{mean}}=87.3\pm 8.7\text{ mmHg}$, Fig. 3c; ${\text{dP}}_{L1}^{max}=47.2\pm 9.5\text{ mmHg}$; ${\text{dP}}_{L2}^{max}=81.3\pm 9.0\text{ mmHg}$; ${\text{dP}}_{L3}^{max}=127.3\pm 11.8\text{ mmHg}$, Fig. 3d), in the peak blood flow velocity (${\text{v}}_{L1}^{max}=3.4\pm 0.3\text{ m}/\text{s}$; ${\text{v}}_{L2}^{max}=4.5\pm 0.3\text{ m}/\text{s}$; ${\text{v}}_{L3}^{max}=5.6\pm 0.3\text{ m}/\text{s}$, Fig. 3e). Further, the energy loss index decreased (ΔELI_*L*1_ = −81.0 ± 0.5 %; ΔELI_*L*2_ = −90.4 ± 0.2 %; ΔELI_*L*3_ = −96.2 ± 2.9 %, Fig. 3f, and the valvulo-arterial impedance increased ($\Delta {\text{Z}}_{L1}^{VA}=22.4\pm 3.0\;\%$; $\Delta {\text{Z}}_{L2}^{VA}=75.1\pm 6.7\;\%$; $\Delta {\text{Z}}_{L3}^{VA}=153.3\pm 24.4\;\%$, Fig. 3g). Akin to the changes in cardiac function (Fig. 2 d–i), changes in each of these metrics were acutely reversed due to debanding, with greater variability in the return state than at baseline, likely due to differences in hemodynamic compensation to pressure overload.

### Platform versatility for various banding and debanding modalities and cardiac chambers

**Extended Data Fig. 2 |:**
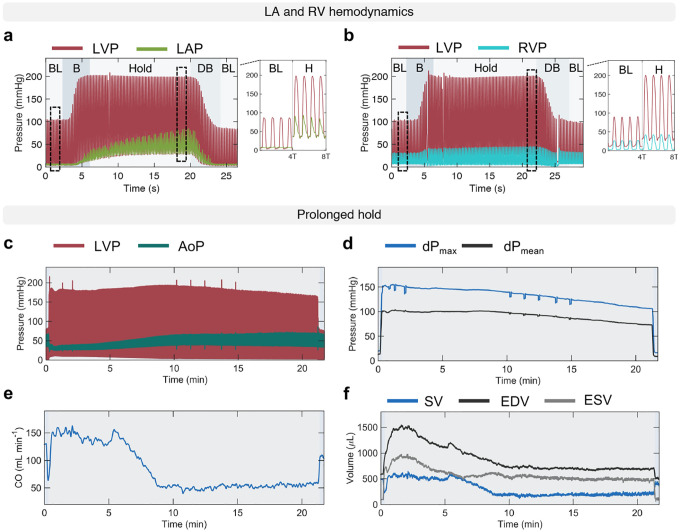
Hemodynamics of the LA and RV and of the LV during a prolonged hold study. **a-b,** Representative LVP and LAP (**a**) and LVP and RVP (**b**) waveforms during aortic banding to L3, 20-second hold, and debanding at L3. Detail of waveforms at baseline and before debanding. **c-f,** LV hemodynamics during a prolonged actuation study, showing LVP and AoP waveforms (**c**), dP_*mean*_ and dP_*max*_ (**d**), CO (**e**), and SV, EDV, ESV (**f**). LVP = left ventricular pressure; LAP = left atrial pressure; RVP = right ventricular pressure; BL = baseline; B = banding; DB = debanding; H = hold; AoP = aortic pressure; dP = transvalvular pressure gradient; CO = cardiac output; SV = stroke volume; EDV = end-diastolic volume; ESV = end-systolic volume.

Extended data Fig. 2 a–b showcases the use of our platform to measure hemodynamic changes in the left atrium (LA) and right ventricle (RV). Aortic banding resulted in an upward shift in the LA pressure (LAP), with increased minimum and maximum LAP, corresponding to emptying into the LV (y-descent) and atrial contraction (a-wave), respectively. Notably, both minimum and maximum LAP increased more slowly than the LVP during the banding phase, with the minimum LAP reaching steady state approximately five seconds into the hold phase, and the maximum LAP continuing to increase for additional ten seconds due to increased atrial filling. At the same level of aortic banding, the peak RV pressure (RVP) increased, while the end-diastolic RVP decreased. Both these changes occurred during the banding phase and remained at steady state during the hold phase, before being reversed due to debanding (extended data Fig. 2b).

**Fig. 4 |:**
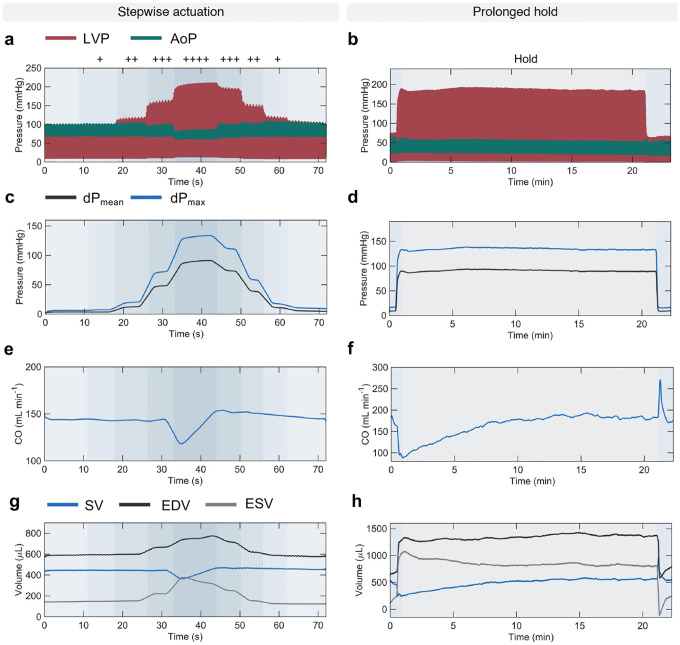
Stepwise and prolonged actuation studies. **a-h,** Evolution of metrics of cardiac function and aortic hemodynamics during a stepwise and prolonged hold actuation studies, showing LVP and AoP waveforms (**a-b**), dP_*mean*_ and dP_*max*_ (**c-d**), CO (**e-f**), and SV, EDV, ESV (**g-h**). Actuation volumes: 0.24, 0.26, 0.28, 0.3 mL, where + indicates progression from one actuation level to another. LVP = left ventricular pressure; AoP = aortic pressure; dP = transvalvular pressure gradient; CO = cardiac output; SV = stroke volume; EDV = end-diastolic volume; ESV = end-systolic volume.

Fig. 4 further demonstrates the control of pressure overload that can be achieved using the soft robotic actuator through stepwise and prolonged hold actuation studies via measurements of LVP and AoP (Fig. 4 a–b), dP_*max*_ and dP_*mean*_ (Fig. 4 c–d), CO (Fig. 4 e–f), and SV, EDV, ESV (Fig. 4 g–h). The stepwise banding and debanding study was conducted in steps of 0.02 mL of actuation medium and showed consistent trends in changes of metrics of LV and aortic hemodynamics, as demonstrated previously (Fig. 2a, 2e, 2g–i, 3c–d). Stepwise banding resulted in progressive elevations in systolic LVP and a drop in AoP (Fig. 4a) and in dP_*mean*_ and dP_*max*_ (Fig. 4c), a progressive drop in CO (Fig. 4e) and SV (Fig. 4g). Changes in LVP and AoP during stepwise debanding were opposite but lesser in magnitude than during banding, likely due to compensation (Fig. 4a,c). Compensatory mechanisms during stepwise actuation can also be observed by the increase in CO and SV at peak banding (Fig. 4 e,g). Stepwise debanding caused the EDV and ESV to return to baseline, fully restoring baseline values of CO and SV (Fig. 4 e,g).

The prolonged hold study was performed to demonstrate the ability of the actuator to remain inflated over time. Fig. 4b shows representative LVP and AoP waveforms over approximately 20 minutes. Associated sustained changes in dP_*mean*_ and dP_*max*_ (Fig. 4d), CO (Fig. 4f), and SV, EDV, and ESV (Fig. 4h) are consistent with findings in the acute study. Further, the gradual increase in CO and SV in Fig. 4 f,h highlights the onset of compensatory mechanisms due to prolonged hold. An additional example of a prolonged actuation study is illustrated in extended data Fig. 2 c–f. These results support the use of our model for controlled, progressive, and reversible aortic constriction in the chronic setting.

### MRI evaluation of proof-of-concept chronic study of progressive pressure overload and reversal

**Extended Data Fig. 3 |:**
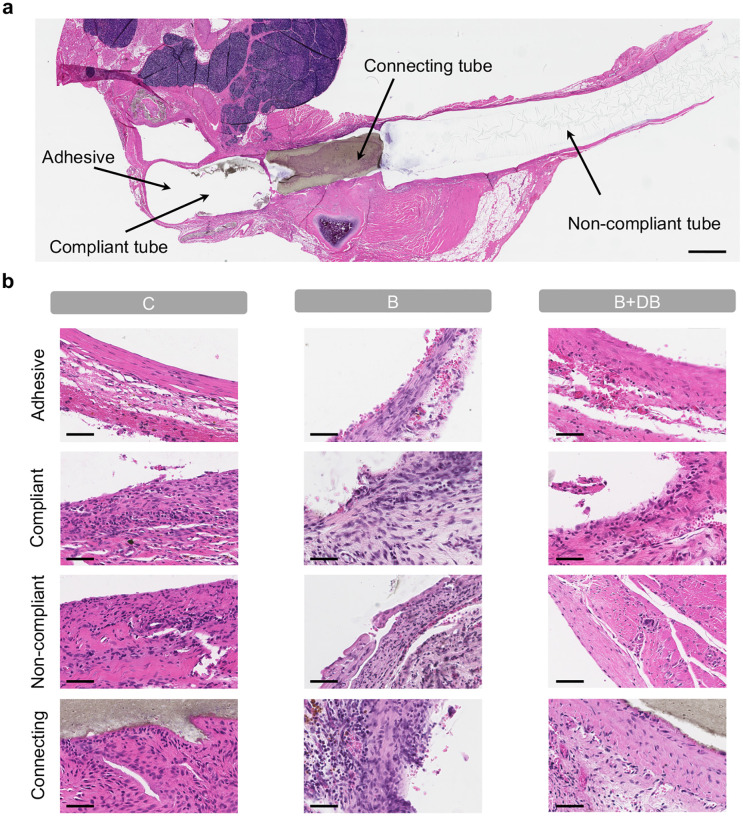
Aortic tissue histology. **a,** H&E image in longitudinal view of the aortic tissue and the implanted soft robotic actuator. Arrows highlight the components of the actuator, including (from left to right) the adhesive, the compliant, the connecting, and the non-compliant tubes. Scale bar = 1.5 mm. **b** Representative H&E images showing unremarkable inflammatory response of the tissue to the adhesive, and of the compliant, non-compliant, and connecting tubes for the control (C), banding (B), and banding-debanding (B+DB) groups. Scale bar = 50 *μ*m.

**Fig. 5 |:**
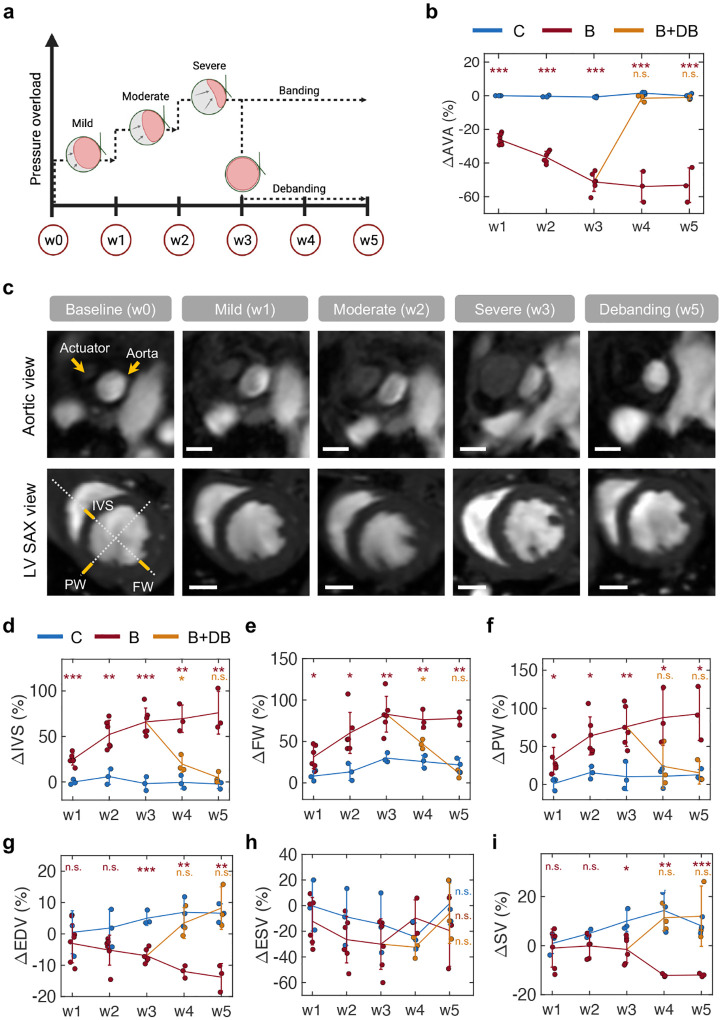
MRI study of chronic cardiac remodeling and reversal via progressive banding and debanding. **a,** Overview of the timeline of the proof-of-concept chronic studies and levels of pressure overload. **b,** Changes in AVA measured weekly throughout the study. **c,** Representative anatomical images of the aorta and LV in short-axis (SAX) view for various levels of banding. Scale bars = 2 mm. **d-f,** LV structural changes over time, including IVS (**d**), FW (**e**), and PW (**f**) thickness. **g-i,** Changes in metrics of LV function over time, including EDV (**g**), ESV (**h**), and SV (**i**). N = 3 in each group; n = 6 in banding group weeks 1–3. *:*P* < 0.05, **:*P* < 0.01, ***:*P* < 0.001, n.s.: non-significant. C = control; B = banding; B+DB = banding-debanding; AVA = anatomic valvular area; SAX = short-axis view; IVS = interventricular septum; FW = free wall; PW = posterior wall; EDV = end-diastolic volume; ESV = end-systolic volume; SV = stroke volume.

We designed a proof-of-concept chronic study to showcase the feasibility of the proposed model for long-term studies of aortic banding and cardiac remodeling as well as of its tunability and versatility. We implanted the actuator in a total of n = 9 Sprague Dawley female rats. In the control group (C; n = 3), the actuator was implanted but never actuated. In the remaining n = 6 rats, the degree of aortic constriction was progressively increased over the course of three weeks. At the three-week time point and for two additional weeks, the maximal level of aortic banding was then maintained in the banding group (B; n = 3), while it was reversed in the banding-debanding group (B+DB; n = 3). The progressive levels of pressure overload in the banding and banding-debanding groups are shown in Fig. 5a. H&E images in extended data Fig. 3 show that chronic implantation of the actuator does not result in any remarkable inflammatory response of the aortic tissue at the five-week time point, supporting the suitability of long-term studies. Changes in LV structure and function in all animals were evaluated weekly via MRI for a total of five weeks post-implantation. Histological analysis of aortic and LV tissue was conducted on sacrifice.

**Extended Data Fig. 4 |:**
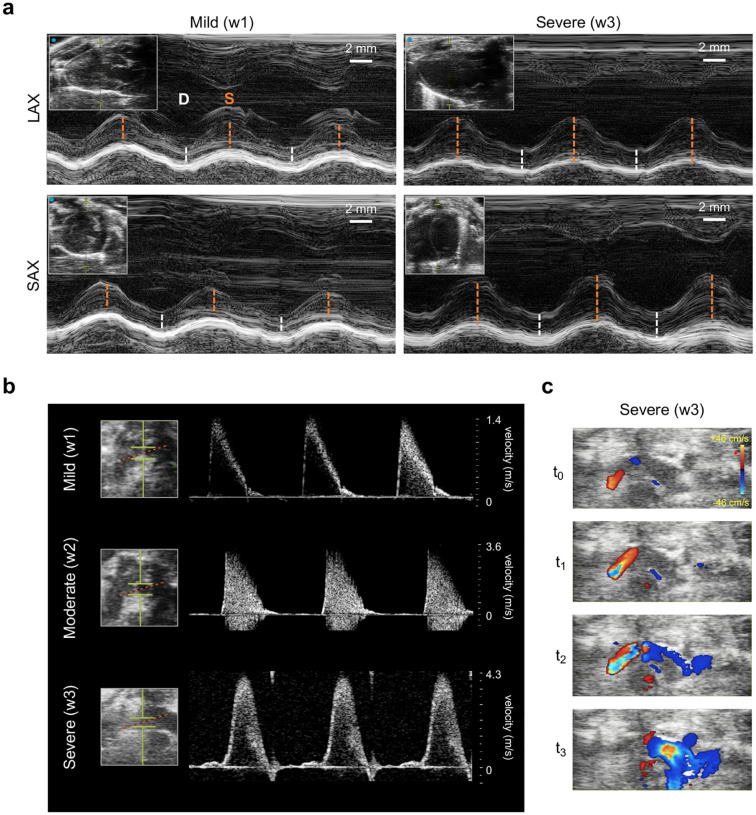
Examples of ultrasound structural and functional characterization of aortic banding model. **a,** Representative M-mode images of the LV in long-axis (LAX) and short-axis (SAX) at mild (w1) and severe (w3) aortic banding. Dashed lines indicate wall thickness during systole (S) and diastole (D). **b,** Representative pulsewave (PW) imaging of blood flow through the soft robotic actuator for mild (w1), moderate (w2), and severe (w3) aortic banding. **c,** Representative color Doppler imaging at four progressive time points (t0-t3) for severe aortic banding (w3). LAX = long-axis view; SAX = short-axis view; D = diastole; S = systole.

Fig. 5b illustrates variations in the aortic cross-section (or anatomical valvular area, AVA) across the three groups. At week three, the actuated groups (B and B+DB) show a reduction in AVA of −51.2 ± 5.6 % (P<0.001). At weeks four and five, this drop is maintained in the banding group ($\Delta {\text{AVA}}_{w4}^{B}=-54.0\pm 9.2\;\%$, P<0.001; $\Delta {\text{AVA}}_{w5}^{B}=-53.1\pm 10.3\;\%$, P<0.001) whereas it is reversed in the banding-debanding group ($\Delta {\text{AVA}}_{w4}^{B+DB}=-1.5\pm 2.0\;\%$, n.s.; $\Delta {\text{AVA}}_{w5}^{B+DB}=-1.0\pm 1.1\;\%$, n.s.). Representative MRI anatomical images of the aortic cross-section in short-axis view (SAX) are shown in Fig. 5c. This highlights the progressive increase in the actuator cross-sectional area and the subsequent drop in aortic cross-section from baseline to severe actuation. These changes are then reversed at week five in the banding-debanding group. At each time point, corresponding SAX images of the LV are also shown. Changes in the inter-ventricular septum (IVS), posterior wall (PW), and free wall (FW) thickness were measured via MRI throughout the study as indicative of LV remodeling due to pressure overload. Fig. 5c highlights the thickening of the LV wall from baseline to week three and reversal to baseline at week five due to debanding. Representative M-mode images of the LV in LAX and SAX, as well as pulse wave (PW) and color-Doppler imaging of blood flow through the actuator obtained using ultrasound at various degrees of aortic banding are shown in extended data Fig. 4.

**Extended Data Fig. 5 |:**
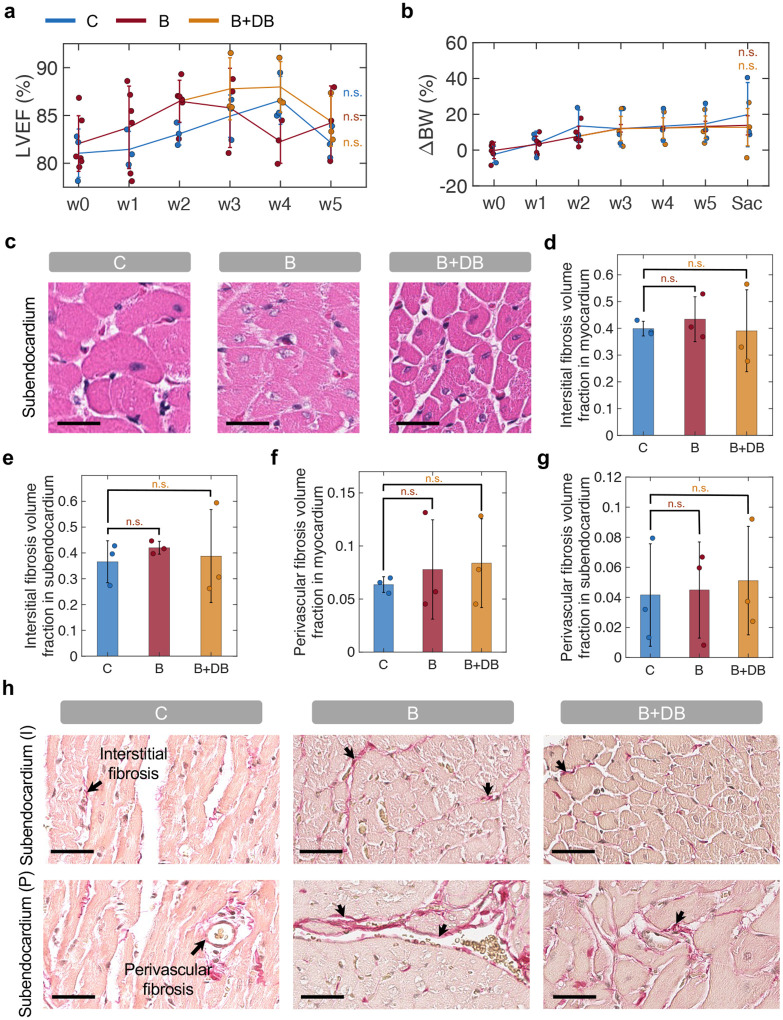
Additional metrics of functional and histology evaluation. **a,** Left ventricular ejection fraction (LVEF). **b,** Changes in body weight (BW) relative to post-op measurements. **c,** Representative H&E sides of the subendocardium. Scale bar = 20 *μ*m. **d-h,** Fibrosis evaluation, showing interstitial volume-weighted mean volume (Vv) in the myocardium (**d**) and subendocardium (**e**), perivascular volume fraction (Vf) in the myocardium (**f**) and subendocardium (**g**), and representative picrosirius red images of interstitial (I) and perivascular (P) fibrosis in the subendocardium (**i**). Scale bar = 40 *μ*m. n.s.: non-significant. C = control; B = banding; B+DB = banding-debanding; LVEF = left ventricular ejection fraction; BW = body weight; I = interstitial; P = perivascular.

Quantitatively, Fig. 5d–f illustrates changes in LV wall thickness throughout the five-week study with respect to baseline. At peak banding, the IVS (Fig. 5d), FW (Fig. 5e), and PW (Fig. 5f) thickness increased significantly in the actuated groups ($\Delta {\text{IVS}}_{w3}^{B}=66.0\pm 15.2\;\%$, P<0.001; $\Delta {\text{FW}}_{w3}^{B}=82.8\pm 21.6\;\%$, P<0.001; $\Delta {\text{PW}}_{w3}^{B}=75.8\pm 25.7\;\%$, P<0.001). In the banding group, these values increased progressively through week five ($\Delta {\text{IVS}}_{w5}^{B}=76.0\pm 23.5\;\%$, P<0.001; $\Delta {\text{FW}}_{w5}^{B}=77.7\pm 7.8\;\%$, P<0.001; $\Delta {\text{PW}}_{w5}^{B}=92.8\pm 35.2\;\%$, P<0.001), while they dropped due to debanding ($\Delta {\text{IVS}}_{w5}^{B+DB}=4.7\pm 6.0\;\%$, n.s.; $\Delta {\text{FW}}_{w5}^{B+DB}=11.4\pm 4.8\;\%$, n.s.; $\Delta {\text{PW}}_{w5}^{B+DB}=15.4\pm 14.9\;\%$, n.s.). At week five, there was no significant difference in LV wall thickness between the control and the banding-debanding groups, indicating that these remodeling processes may be reversible if the pressure overload is relieved. Fig. 5 g–i shows that progressive aortic banding results in a progressive drop in EDV ($\Delta {\text{EDV}}_{w3}^{B}=-7.0\pm 2.1\;\%$, P<0.001; $\Delta {\text{EDV}}_{w5}^{B}=-13.8\pm 4.3\;\%$, P<0.001) (Fig. 5g). Because aortic banding did not cause significant changes in ESV ($\Delta {\text{ESV}}_{w5}^{C}=1.5\pm 16.4\;\%$, n.s.; $\Delta {\text{ESV}}_{w5}^{B}=-19.5\pm 27.9\;\%$, n.s.; $\Delta {\text{ESV}}_{w5}^{B+DB}=-6.1\pm 23.5\;\%$) (Fig. 5h), the SV dropped significantly in these groups ($\Delta {\text{SV}}_{w3}^{B}=-1.5\pm 5.4\;\%$, P<0.001; $\Delta {\text{SV}}_{w5}^{B}=-12.0\pm 0.7\;\%$, P<0.001) (Fig. 5i). In the banding-debanding group, these changes are reversed ($\Delta {\text{SV}}_{w5}^{B+DB}=12.0\pm 12.3\;\%$, n.s.). No statistically significant change in left ventricular ejection fraction (LVEF) was observed between the start and end of the study in any group (extended data Fig. 5a).

### Evaluation of progressive pressure overload and reversal in chronic model at 6 weeks

**Fig. 6 |:**
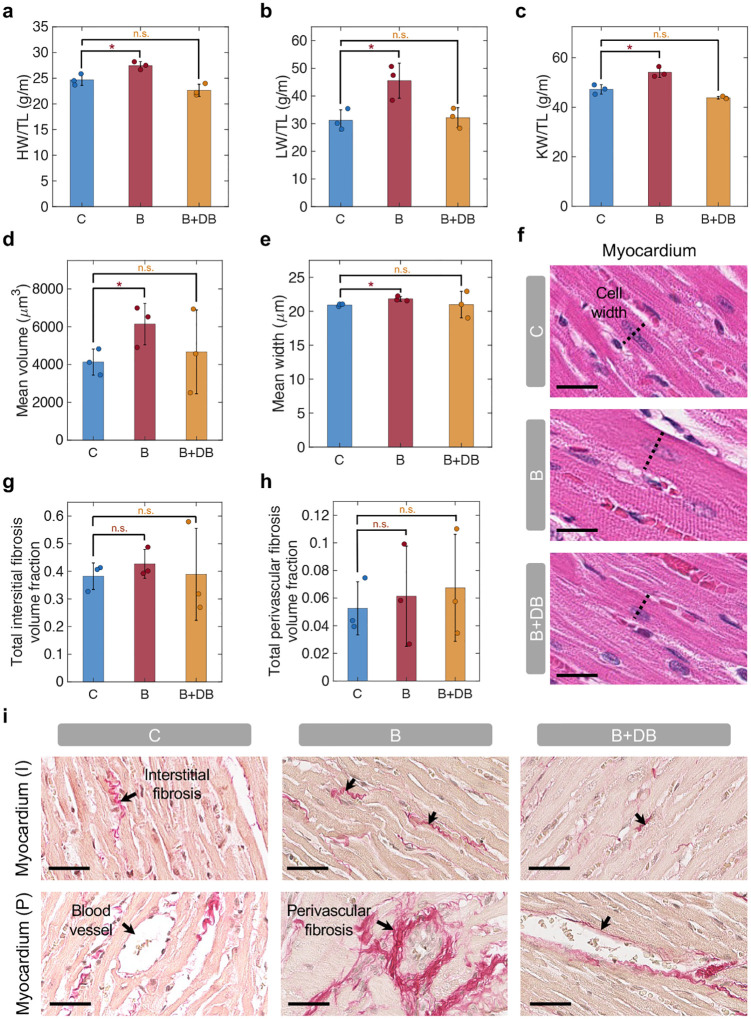
Organ weight measurements and histology analysis. **a-c,** Organ weight to tibial length (TL) ratio, including heart (H) (**a**), lung (L) (**b**), and kidney (K) (**c**). **d-f,** LV cardiomyocyte analysis, showing volume-weighted mean volume (Vv) of cardiomyocytes in the myocardium (**d**), mean cell width in subendocardium (**e**), and representative myocardium H&E slides (**f**). Scale bar = 20 *μ*m. **g-i,** Fibrosis evaluation, showing total interstitial (**g**) and perivascular (**h**) fibrosis volume fraction (Vf), and representative picrosirius red images, highlighting myocardial interstitial (I) and perivascular (P) fibrosis (**i**). Scale bar = 40 *μ*m. N = 3 in each group; n = 6 in banding group weeks 1–3. *:*P* < 0.05, n.s.: non-significant. C = control; B = banding; B+DB = banding-debanding; HW/TL = heart weight to tibial length ratio; LW/TL = lung weight to tibial length ratio; KW/TL = kidney weight to tibial length ratio; I = interstitial; P = perivascular.

Gross anatomic and histologic examination of key organs were conducted after animal sacrifice. We evaluated the heart (HW), lung (LW), and kidney weight (KW) to tibial length (TL) ratios across the three groups (Fig. 6 a–c). The HW/TL (Fig. 6a), LW/TL (Fig. 6b), and KW/TL (Fig. 6c) were moderately increased in the banding group compared to control (HW/TL^*C*^ = 24.7 ± 1.1 g/m, HW/TL^*B*^ = 27.5 ± 0.8 g/m, P<0.05; LW/TL^*C*^ = 31.2 ± 3.8 g/m, LW/TL^*B*^ = 45.5 ± 6.3 g/m, P<0.05; KW/TL^*C*^ = 47.2 ± 1.9 g/m, KW/TL^*B*^ = 54.1 ± 2.1 g/m, P<0.05) while no statistically significant difference was observed between the banding-debanding group and the control (HW/TL^*B*+*DB*^ = 22.7 ± 1.2 g/m, n.s.; LW/TL^*B*+*DB*^ = 31.2 ± 3.6 g/m, n.s.; KW/TL^*B*+*DB*^ = 43.8 ± 0.5 g/m, n.s.). Changes in body weight (BW) during the study indicated no significant difference between the groups (extended data Fig. 5b).

As a result of banding, the mean cardiomyocyte volume-weighted mean volume (Vv) in the myocardium (Fig. 6d) and the mean cell width in the subendocardium (Fig. 6e) were elevated compared to control (Vv^*C*^ = (4.1 ± 0.7) × 10^3^*μm*^3^, Vv^*B*^ = (6.1 ± 1.1) × 10^3^*μm*^3^, P<0.05; mean width^*C*^ = 20.9 ± 0.2 *μm*, mean width^*B*^ = 21.8 ± 0.4 *μm*, P<0.05). Consistent with changes in LV structure and function measured on MRI (Fig. 5 d–i) and with measurements of organ weight (Fig. 6 a–c), these changes in cardiomyocyte volume and width were partially reversed in the B+DB group (Mean volume^*B*+*DB*^ = (4.7 ± 2.2) × 10^3^*μm*^3^, n.s.; Mean width^*B*+*DB*^ = 21.0 ± 2.0 *μm*, n.s.). Representative H&E images of the myocardium and subendocardium in the three groups are shown in Fig. 6f and extended data Fig. 5c, respectively.

Measurements of the total interstitial (Fig. 6g) and perivascular (Fig. 6h) fibrosis volume fraction (Vf) show a trend towards increased fibrosis in the banding group compared to control (total interstitial fibrosis Vf^*C*^ = 0.38 ± 0.05; Vf^*B*^ = 0.43 ± 0.05, n.s; Vf^*B*+*DB*^ = 0.39 ± 0.17, n.s; total perivascular fibrosis Vf^*C*^ = 0.05 ± 0.02; Vf^*B*^ = 0.06 ± 0.04, n.s; Vf^*B*+*DB*^ = 0.07 ± 0.04, n.s), although these differences did no reach statistical significance. Measurements of interstitial and perivascular fibrosis in the myocardium and subendocardium, individually, show analogous trends (extended data Fig. 5 d–g). Representative images of the myocardium (Fig. 6i) and subendocardium (extended data Fig. 5h) stained with picrosirius red illustrate differences in the interstitial and perivascular fibrosis across the three groups.

## Discussion

This works presents the development of a highly tunable small animal model of LV pressure overload based on an implantable soft robotic actuator. By relying on an actuatable material that can achieve controlled, progressive, and reversible aortic banding in a rat model, this platform overcomes several of the limitations associated with other preclinical models of disease, such as lack of tunability of the degree of pressure overload and the subsequent inability to recapitulate the natural progression of disease or reversal due to medical intervention.

The soft actuator is composed of a compliant tube that expands under pressure (Fig. 1a). Its expansion can be controlled by tuning the actuation volume, which results in predictable changes in the diameter and pressure of the actuator (Fig. 1 c–e). In terminal studies, we used invasive hemodynamic monitoring to measure metrics of cardiac function (Fig. 2) and aortic hemodynamics (Fig. 3), during progressive banding, debanding, and for three discrete actuation levels. We obtained LV PV loops (Fig. 2c) and measured LVP_*max*_ (Fig. 2d), CO (Fig. 2e), SW (Fig. 2f), and LV volumes (Fig. 2 g–i). We also calculated diagnostic metrics of AS, such as EOA (Fig. 3b), dP (Fig. 3 c–d), v_*max*_ (Fig. 3e), ELI (Fig. 3f), and Z_*V A*_ (Fig. 3g). We characterized changes in LV and aortic hemodynamics during stepwise and a prolonged actuation study (Fig. 4) and in LA and RV pressure due to aortic banding and debanding (extended data Fig. 2). In addition to being consistent with the hemodynamics of pressure overload, changes in these metrics demonstrated the ability of the system to induce various levels of aortic banding and to return to the original hemodynamic state via debanding, which cannot currently be simultaneously achieved by any of the existing small animal models of pressure overload.

Although many other models of aortic banding rely on ultrasound imaging to characterize hemodynamics of pressure overload [16, 18, 22, 23, 26, 33–36], in this work, we leveraged MRI to directly visualize the degree of aortic constriction due to the soft actuator. Nevertheless, the proposed model remains compatible with ultrasound imaging as demonstrated in extended data Fig. 4. Albeit more expensive and time-consuming, MRI allows for direct measurements of aortic constriction and LV remodeling, while also reducing measurement variability due to operator experience associated with echocardiography [37]. Further, MRI allowed us to visualize blood flow through the aortic constriction (Fig. 3a, extended data Fig. 1), which could be key in future studies of shear and vortex formation and their impact on disease progression [32].

We then demonstrated the potential of the proposed disease model for long-term studies of pressure overload through a proof-of-concept five-week investigation. The actuator was used to achieve progressive aortic banding, through three distinct levels, over the course of three weeks. This level of pressure overload was then maintained for two additional weeks in a subset of the animals in the study, while it was reversed to baseline in another subset (Fig. 5a). Weekly MRI studies were used to visualize and quantify the degree of aortic constriction (Fig. 5b) as well as structural changes in the LV due to remodeling (Fig. 5 c–f). Consistent with the literature, which reports a 30–60% increase in the IVS thickness and approximately a 50% increase in PW thickness at five to eight weeks post-banding [16, 35], our model resulted in a 66–76% and 76–93% increase in IVS and PW thickness, respectively (Fig. 5 d,f). In our work, these changes occurred three to five weeks post-debanding, that is two to three weeks sooner than in these other studies, likely due to variations in the levels of aortic constriction achieved. In addition, the ability to perform debanding suggests that after three weeks of banding, these changes in LV wall thickness are almost completely reversible (80–90% reduction) two weeks post-debanding (Fig. 5d,f). Other studies have shown analogous results, with a 60–70% drop in IVS thickness two weeks post-debanding after five weeks of banding [35]. However, all the previous investigations of reversal of cardiac remodeling in small animals required a second surgery to invasively remove the aortic clip or suture [25, 28–30], whereas our platform does not require any additional intervention to deband the aorta.

These findings were consistent with the changes in cardiac function measured via MRI, organ weight, and histology. Thickening of the LV resulted in a progressive (and reversible) drop in the EDV (Fig. 5g) and SV (Fig. 5i), while no changes in contractility were observed during this study, as elucidated by measurements of ESV (Fig. 5h) and LVEF (extended data Fig. 5a). Measurements of organ weight showed a moderate increase in the HW/TL (Fig. 6a), LW/TL (Fig. 6b), and KW/TL (Fig. 6c) in the banding group compared to control. Further, histological findings highlighted an approximately 50% increase in the mean cardiomyocyte volume (Fig. 6d) due to aortic banding. Analogously, the total (myocardial and subendocardial) interstitial (Fig. 6g) and perivascular (Fig. 6h) Vf increased roughly by 12% and 17%, respectively, in the banding group. Interestingly, perivascular fibrosis did not exhibit significant reversal due to debanding, which is likely because of potential differences in the time needed for it to resolve, compared to reversal of interstitial fibrosis and cardiomyocyte size.

While overcoming the inability to control the degree of aortic banding, there are some limitations of this study that should be discussed. First, implantation of a soft robotic actuator around the ascending aorta of a small animal is extremely challenging, and it requires excellent surgical skills. The additional difficulties and the time required for the surgery compared to simple aortic ligation may result in elevated post-operative mortality rates. However, the ability to control for the degree of pressure overload may enable partial debanding that could improve animal recovery. Secondly, although this work aims to demonstrate the feasibility of chronic studies of pressure overload and LV remodeling, a more comprehensive characterization of various aortic banding states remains to be conducted. Finally, this work did not investigate the degree of reversibility of remodeling processes for various levels of aortic constriction and debanding duration. These studies could shed light on ventricular plasticity and the optimal time of intervention to halt or reverse remodeling, restoring adequate cardiac mechanics and hemodynamics. Clinically, they could ultimately impact current management strategies for patients with cardiac remodeling secondary to AS or hypertension [38–40].

Models of aortic banding have been developed to combine pressure overload with metabolic conditions or other comorbidities, including obesity or diabetes, that are often associated with HFpEF [41–43]. In addition, several studies report diminished cardiac contractility and the transition from HFpEF to heart failure with reduced ejection fraction (HFrEF), often occurring 7–21 weeks after aortic constriction [15, 19]. In future work, we aim to investigate the integration of the proposed model with current strategies to co-induce HFpEF comorbidities, as well as the long-term modulation of pressure overload to mitigate the risk of transition to phenotypes other than HFpEF. If successful, these studies could further elucidate the interplay between mechanical and biological stressors and further contribute to the establishment of a more clinically relevant small animal model of HFpEF.

Ultimately, the ability to recapitulate and control multiple aspects of disease may lend us towards the development of increasingly more accurate models of human physiology and disease. These models could play a critical role in allowing us to elucidate mechanisms of disease not yet fully understood and develop more effective treatments for patients affected by these conditions. As the first application of soft robotics for the development of a disease model in small animals, this work may pave the way towards the design of highly tuneable models of disease within and beyond the cardiovascular space, providing a new toolset to improve current methods for fundamental and preclinical research.

## Methods

### Actuator characterization

The mechanical properties of the materials constituting the soft robotic actuator were measured under uniaxial tensile loading using a 2 kN load cell on an an electromechanical tester (Instron 5566, MA). A strain rate of 0.05 s^−1^, corresponding to 0.75 mm/s for a gauge length of 15 mm, was used for testing. The fabric sheet was cut to a width of 2.7 mm. The sheet had a thickness of 0.2 mm and the tubing preserved original inner diameter (ID) and outer diameter (OD) dimensions. Uniaxial tensile testing was conducted to failure on n = 5 specimens for each material. The raw force-displacement data were converted to engineering stress-strain using equations 1–2.

(1) $$\sigma =\frac{F}{A};$$

(2) $$\epsilon =\frac{l-l_{0}}{l_{0}};$$

where *σ* and *ϵ* are the engineering stress and strain, *F* and *l* are the force and the displacement applied to the sample, and *A* and *l*_0_ are the initial cross-sectional area and length of the sample, respectively. The Young’s modulus *E* was calculated as the slope of the stress-strain curve in the *ϵ* = 0 – 0.5 interval. Data were plotted up to failure point.

Changes in the pressure and expansion of the actuator during banding and debanding were measured by deploying up to 0.3 mL of medium (glycerin, *ρ* = 1.26 g/cm^3^; *ν* = 11.10 mPa s) using a syringe pump (70–3007 PHD ULTRA Syringe Pump Infuse/Withdraw, Harvard Apparatus, MA) at a constant rate of 0.03 mL/s. Volumes of 0.26, 0.28, and 0.3 mL were infused during testing at discrete actuation levels, corresponding to levels L1-L3. The pressure inside the actuator was measured using the PS-3203 wireless pressure sensor (PASCO, CA) connected to the actuator via a three-way valve. Pressures were recorded continuously using Capstone software (v.2.2.0, PASCO). Changes in diameter were recorded using a camera oriented perpendicularly to the actuator to capture the maximal diameter during expansion. Images were processed using ImageJ for Mac OS X.

### Implantation for in vivo studies

All animal procedures were reviewed and approved by the Institutional Animal Care and Use Committee (IACUC) of the Massachusetts Institute of Technology. In this study, we used Sprague-Dawley female rats (BW = 241.8 ± 11.5 g). Before surgery, all animals were anesthetized using 2–4% Isoflurane per O2 in effect following weighing, sterile eye ointment was applied, and the back and the chest of the animals were shaved. For survival studies, preoperative antibiotic and analgesic medications were administered, including Enrofloxacin (10mg/kg, IM), Buprenorphine (1.0–1.2 mg/kg, SQ), Meloxicam (2 mg/kg, SQ), Lidocaine (5–10 mg/kg, SQ). All surgical tools were autoclaved prior to survival surgeries. Warmed saline (1 mL) was administered preoperatively SQ. Endotracheal intubation was then performed to allow mechanical ventilatation (2.5 ml per breath at 70 breaths per minute). Temperature support was provided with an electric heating pad during the surgery.

The soft robotic actuator was implanted via sternotomy or left thoracotomy at the fourth intercostal space for terminal and survival procedures, respectively. Careful dissection of the muscle layers was performed before placement of a self-retaining retractor to allow better visualization of and access to the ascending aorta. The ascending aorta could then be identified and carefully isolated. The actuator was therefore wrapped loosely around the ascending aorta and then tightened by creating a double knot with the fabric sheet. The non-compliant tubing was tunneled out of the chest wall through a small incision in the third intercostal space and into the lateral subcutaneous space. The intercostal spaces were then closed using several interrupted 4–0 Vicryl sutures. A fenestrated flexible 24G catheter connected to a 5 mL syringe was inserted in the chest cavity through the thoracotomy and suction was applied to restore negative pressure in the thorax during a breath-hold before the final suture was tied town. The inflexible tubing was tunneled above the left forelimb to the left anterior chest above the forelimb. A counter incision was made in the skin in preparation for tunneling to the dorsum and the port for the tubing secured in the subcutaneous tissue. The subcutaneous and skin layers of the thoracotomy incision were then closed with Vicryl as above.

For placement of the percutaneous port for survival surgeries, the rat was turned to the prone position and a small incision (1–2 cm) was made between the shoulder blades of the animal after sterile preparation of the surgical site. The tubing of the device was grasped with forceps through the lateral counter incision and then tunneled through the dorsal side. An MRI-safe vascular access button (VAB62SMBS/25-MRI or VAB95BS-MRI, Instech Laboratories, PA) was connected to the distal end of the tubing and placed subcutaneously between the shoulder blades of the rat. The tubing was finally secured with a 5–0 nylon suture to the port. The skinincisions were then closed with 5–0 nylon and skin glue.

After the procedure, the isoflurane was decreased to 1% and the ventilator was turned off while the animal was monitored for spontaneous breathing. Following return to normal respiration, the isoflurane was turned off. The animals were then extubated. For survival procedures, Meloxicam (2 mg/kg, PO) and enrofloxacin (2mg tablets, PO) were administered at 24 hr and 48 hr post-surgery.

### Cardiac and aortic catheterization

For terminal catheterization, a median sternotomy was used for the surgical approach as detailed above. Invasive hemodynamic measurements were obtained using two 2F straight PV catheters (SPR-838, Millar, TX) that were inserted after puncture with a 27G needle. The catheters were introduced into the LV for LV, aortic, and LA monitoring, and through the RV for RV monitoring. When needed, one catheter was then advanced through the aortic or the mitral valve for aortic and LA monitoring, respectively. The catheters were connected to the MPVS Ultra console (Millar, TX), which was in turn connected to the Powerlab system (PowerLab 8/26, ADInstruments, Australia) for monitoring and acquisition. The data were displayed and recorded in real time on LabChart (Pro v8.1.16, ADInstruments). A sampling frequency of 1000 Hz and a bandstop filter at 50Hz were used.

LV and aortic characterization was conducted on n = 5 rats. RV and LA hemodynamics, LV and aortic hemodynamics during prolonged actuation, and LV and aortic hemodynamics during stepwise actuation were measured on a subset of n = 3 rats per group. Steps of 0.01–0.02 mL of medium were used to progress from one actuation level to the other (L1-L3) for each study, using the 70–3007 PHD ULTRA Syringe Pump (Harvard Apparatus) at a rate of 0.03 mL/s. Following invasive hemodynamic evaluations, the rats were sacrificed by aortic transection under anesthesia.

### Calculation of AS hemodynamic metrics

Metrics of AS during catheterization studies were calculated using standard equations. The Gorlin equation was used to compute the EOA (Equation 3) [44]:

(3) $$\text{iEOA}=\frac{Q}{\text{BSA}*51.6*\sqrt{\Delta P_{\text{mean}}}}$$

where *Q* is the aortic flow, BSA is the body surface area of the animal, and ΔP_*mean*_ is the mean pressure gradient, as measured with the LV and aortic catheters.

v_*max*_ was estimated from PV measurements of ΔP_*max*_ (Equation 4) [45].


(4) $$v_{max}=\sqrt{\frac{\Delta P_{\text{max}}}{4}}$$

ELI was calculated using Equation 5 [46]:

(5) $$\text{ELI}=\frac{\text{EOA}\left(A_{\text{a}}\right)}{\text{BSA}\left(A_{\text{a}}-\text{EOA}\right)}$$

where and A_*a*_ is the cross-sectional area of the aorta measured at the sinotubular junction. Z_*V A*_ was measured using Equation 6 [47]:

(6) $$Z_{\text{VA}}=\frac{{\text{LVP}}_{\text{max}}}{\text{SV}}\text{BSA}$$

where LVP_*max*_ is the peak systolic LV pressure and SV is the stroke volume, as obtained from LV catheterization.

In these equations, BSA was estimated from the body weight (BW) of the rat using the Rubner formula [48] (Equation 7):

(7) $$\text{BSA}=9.46{\text{BW}}^{\frac{2}{3}}$$

### MRI data acquisition

MRI was performed in vivo on a 7T MRI operated by Bruker AV4 NeoBioSpec70-20USR console, equipped with a 114 mm 660 mT/m actively shielded gradient (Bruker BioSpin, Rheinstetten, Germany) and a 63 mm inner diameter Varian volume RF coil (Agilent Technologies). Rats were anesthetized by inhalation of 3% isofluorane and maintained on 2–2.5% isofluorane throughout data collection. Body temperature, respiration rate, and the EKG signal were observed via the SAII monitoring and gating system (Small Animal Instruments Inc., Stony Brook). All data were collected and reconstructed within Bruker Paravision PV360 v2.0. Images were converted to DICOM format and subsequently analyzed.

Slice position was determined based on the *Localizer* and *Planning* protocols. Quantitative flow maps of the aorta were acquired using the *Velocity Map* protocol with the following parameters: TE/TR = 5/30 ms, 3 averages, and a flip angle of 40° in the slice direction. The velocity encoding (VENC) value was adjusted based on estimated aorta flow rates, ranging from 110 to 500 cm/s. A FOV of 40 × 40 mm, in-plane data matrix 256 × 256, and a slice thickness of 0.7 mm with no gap were used for this study. Triggering was turned on per slice for both EKG and respiratory gating.

SAX and LAX images of the LV were acquired using the *Cine Brightblood IG FLASH* protocol. Sixteen CINE time frames were collected to ensure the acquisition of an entire cardiac cycle. Stacks of 2D slices resulted in a resolution of 0.26 × 0.26 × 1 mm. Fast anatomies of the aorta were collected using the *Planning* protocol with TE/TR = 3/45 ms, 18 averages, and a flip angle of 35° to minimize imaging time, providing a spatial resolution of 0.156 × 0.156 × 0.7 mm.

### MRI data processing

The processing of 2D-PCMRI data was performed on Matlab 2020a (Mathworks, MA). Analysis involved vessel segmentation, noise masking, velocity anti-aliasing, and spatial interpolation (extended data Fig. 1a). Masking was executed by utilizing the raw data magnitude within the region where the ascending arterial structure and the actuator were apparent on the imaging plane. Velocity vectors were then calculated from the VENC dataset (VENC, 16-bit), utilizing the phase data (*d*_*mag*_), denoting the velocity through the slice-plane direction, per Equation 8:

(8) $${\text{v}}_{\text{mag}}=\left(\frac{{\text{d}}_{\text{mag}}}{65536}\right)\text{VENC}$$

The mask was superimposed to the phase data onto the segmented borders, improving flow visualization and quantification. Aliasing correction was achieved by unwrapping of phase data and the detection of temporal jumps greater than ±*π* at a local level. The baseline for the aliasing correction was established using diastolic blood flow velocity. The velocity magnitude data was converted into the point cloud using the Visualization Tool Kit (VTK) libraries (VTK, Kitware, Inc., NY). The point cloud was then imported into a 3D flow visualization software application (ParaView 5.9., Kitware Inc., Los Alamos National Labs). Color-coded velocity field was used to depict the flow velocity through each plane at different levels of aortic constriction.

OsiriX MD (v.13.0.1, Switzerland) was used to measure changes in AVA and LV wall thickness and volume weekly for five weeks. The AVA was computed as the cross-sectional aortic area on the slice of maximal actuator inflation. LV wall thicknesses were measured in the SAX LV mid-section during end-diastole. The IVS and FW were identified by drawing a line bisecting both ventricles, and a line perpendicular to this was then drawn for PW thickness calculation. LV volumes (LVV) were obtained by measuring the LV area (LVA) in SAX and the LV length (LVL) in LAX during peak-systole and end-diastole per the hemisphere-cylinder model (Equation 9) [49]. Statistical significance was determined with respect to control using two-tailed t-tests with a 95% confidence interval (P<0.05) on MATLAB R2020a. Statistical analysis on ESV and LVEF was conducted within each group longitudinally between the start and the end of the study.


(9) $$\text{LVV}=\frac{5}{6}*\text{A}*\text{L}$$

### Ultrasound evaluation

Transthoracic ultrasound evaluation of aortic flow and LV anatomy was conducted on a rat-handling platform (FUJIFILM Sonosite, Toronto, Canada) using a Vevo 3100 imaging system (FUJIFILM Sonosite, Toronto, Canada). This was equipped with an MX 201 linear array transducer (FUJIFILM Sonosite, Toronto, Canada) that was attached to a stereotactic mounting system. 2–4% isoflurane was provided to keep the animals under general anesthesia and the THM1500 Physiological Monitoring Unit was used to track the animal’s EKG and respiratory signals during the study.

B-mode and M-mode images of the LV in parasternal long axis (PLAX) and SAX were acquired for analysis. B-mode SAX images were used to quantify LVA and the IVS, PW and FW thickness, whereas LAX images were obtained to measure LVL. Pulse wave and color Doppler imaging was performed to measure the peak flow velocity (*v*_*max*_) and visualize flow. The position of the probe had to be adjusted to account for variations in the anatomy due to the surgical procedure and the presence of any scar tissue that would compromise the quality of the images. As a result, the correction angle of the PW Doppler sometimes exceeded 45°. All images were processed using the Vevo Lab 5.6 software (FUJIFILM Visualsonics, Canada).

### Histology

All animals were sacrificed two to three days after final MRI evaluation, using a CO_2_ euthanasia chamber and aortic transection. The weight of the heart, lung, and kidneys, and the tibial length were measured using a microbalance and a caliper, respectively. The aortic and LV tissues were preserved in formalin for histological analysis. Slices were cut at a 400 *μ*m gap. Analysis of the aortic tissue was conducted to assess the inflammatory response the implanted actuator using H&E staining. The mid-section of the LV was preserved to quantify changes in cardiomyocyte area and the degree of interstitial and perivascular fibrosis, using H&E and picrosirius red stains. Tissue sectioning and staining was performed by the Histology Core of the Koch Institute for Integrative Cancer Research and the Massachusetts Institute of Technology. For all the histology data, statistical significance was determined with respect to control using two-tailed t-tests with a 95% confidence interval (P<0.05) on MATLAB R2020a.

### Cardiomyocyte size analysis

For calculation of the volume-weighted mean volume (Vv) of myocardial cardiomyocytes, ten non-overlapping 20X H&E images were taken per sample in the myocardium. Two stereological grids (horizontal lines and crosses) were superimposed onto the images (7500 *μ*m^2^), using ImageJ, while ensuring that the scale was previously calibrated consistently for all images. The grid size was determined by taking an average length squared of the cardiomyocytes over approximately 30 images. Using point sampled intercepts, widths of any cardiomyocyte that intersected with the cross were measured along the horizontal line. The Vv of cardiomyocytes in the myocardium was therefore estimated per Equation 10 [50]:

(10) $$\text{Vv}=\left(\frac{\pi }{3}\right){\text{width}}^{3}$$

Twenty 20X images of the subendocardium were taken from H&E stained sections to compute the cardiomyocyte width in the subendocardium. Images were analyzed using the CmyoSize plugin on ImageJ. This fully automated plugin standardizes cardiomyocyte size by filtering for cells that are regular and round in shape and that contain a central round nucleus and visible border [50, 51].

### Interstitial and perivascular fibrosis evaluation

Systematic random sampling and point counting was used to estimate volume fraction (Vf) of interstitial fibrotic tissue to total interstitial space. Ten 20X images were taken in the myo- and subendo- cardium in picrosirius red-stained sections. A random-offset grid (2500 *μ*m^2^) was superimposed onto the images. Interstitial fibrosis was estimated using per Equation 11 [52, 53]:

(11) $$\text{Vf}=\frac{\text{Points (fibrotic tissue)}}{\text{Points (interstitial space)}-\text{Points }(\text{fibrotic blood vessels)}}$$

A second count was performed on the same images to estimate the perivascular fibrosis using Equation 12 [52, 53]:

(12) $$\text{Vf}=\frac{\text{Points (fibrotic blood vessels)}}{\text{Points (interstitial space)}}$$

## Extended Data
